# Developing a Fundamental Theoretical Definition for Athletic Injury: Metaphysics, Logic, and Mathematics

**DOI:** 10.1007/s40279-026-02418-3

**Published:** 2026-05-02

**Authors:** Judd T. Kalkhoven

**Affiliations:** School of Health Sciences, Western Sydney University, Campbelltown Campus, Narellan Rd and Gilchrist Dr, Campbelltown, Sydney, NSW 2560 Australia

## Abstract

Athletic injury remains inadequately conceptualised and poorly defined. Existing definitions lack the conceptual clarity and logical coherence required to support its maturation into a scientifically meaningful and reliably investigable concept. Related constructs that are often integrated into various operational definitions, such as pain and athlete availability, are frequently conflated as fundamental criteria, producing conceptual instability through semantic vagueness, category conflation, and logical contradiction. These deficiencies in conceptual understanding have hindered the development of precise theoretical and operational frameworks capable of supporting formalisation and have, more broadly, undermined the critical scientific principles of predictability, testability, falsifiability, and reproducibility. To address this, this article employs a systematic process of metaphysical analysis and Carnapian explication, grounded in Aristotelian essentialism and classical logic, to develop a new, scientifically robust theoretical definition of athletic injury. This approach utilises well-established logico-philosophical tools such as thought experiments, boundary tests, and deductive reasoning to evaluate the conceptual coherence of existing definitions, and to establish a set of necessary and sufficient conditions for an athletic injury to exist. Through this process, commonly conflated concepts (*Symbebekós*, ‘accidental properties’) are examined for logical independence and disentangled from essential properties (To ti ēn einai, ‘what it is to be’), revealing the logical structure that anchors this construct in observation and enables its expression within a coherent, logico-mathematical predictive framework. The outcome is an integrative framework that aligns the theoretical, observational, and mathematical dimensions of athletic injury and associated constructs, such as injury severity, recovery, and rate of recovery, into a unified, formalised system of mathematically defined and interrelated entities for consistent application in mathematical modelling, including prediction, simulation, and causal inference. This paves the way for advancements in the assessment and modelling of athletic injury and related phenomena.

## Key Points

**Table:** 

Clear, well-defined concepts are essential for scientific inquiry, enabling effective theory formation, operational precision, and adherence to the core scientific principles of predictability, testability, falsifiability, and reproducibility. Athletic injury remains inadequately conceptualised and is frequently applied in vague or contradictory ways, obscuring theoretical clarity, undermining modelling efforts, and eroding predictive accuracy.
To address this, the article employs a systematic process of metaphysical analysis and Carnapian explication. Carnapian explication is a logical method used for transforming vague or pre-scientific concepts into precise, empirically meaningful constructs. Applied here, it provides the conceptual and logical basis for developing a coherent and scientifically robust theoretical definition of athletic injury, and for aligning the theoretical (definitional and logical), observational (lesion formation and progression, mechanical degradation, tissue failures), and mathematical (modelling) dimensions of this construct. This alignment establishes the foundation for its formalisation and operationalisation as a logico-mathematical construct suitable for prediction, simulation, and causal inference.
The transformation of athletic injury into a logico-mathematical construct also establishes the foundation for a formalised and mathematised ontological framework and semantic network. Here, athletic injury and related constructs, such as injury severity, recovery, and rate of recovery, are systematically defined through precise mathematical relationships, establishing consistent quantitative meanings and integration across models, thereby enhancing the clarity, coherence, and predictive reliability of athletic injury research moving forward.
For a more detailed outline of the article’s key contributions and advancements, a comprehensive summary (Table 6) is presented near the end of the paper, integrating the major theoretical, logical, and mathematical developments discussed throughout.

*“Hitting on the direct definition of a concept, though often an essential contribution to progress, remains a preliminary to the discovery of mathematical truths”* – Michael Dummett [1].

*“In any special doctrine of nature, there can be only as much proper science as there is mathematics therein”* – Immanuel Kant [2].

## Introduction

In sports science and medicine, a variety of theoretical definitions (Table 1) of athletic injury have been proposed [3–6], yet none are sufficiently coherent and robust [7]. This is problematic. Just as an axiom provides the foundations for a logical or mathematical system, a theoretical definition establishes the conceptual framework within which a concept can be understood scientifically [8–13]. Certainly, theory-driven research, a fundamental component of the scientific method [7, 10, 14–18], relies upon precise, logically consistent, and empirically testable concepts to explain or predict phenomena [10, 12, 13, 19–21]. Without a sufficiently coherent definition, the theoretical and methodological formalisation (Table 1) of athletic injury and associated constructs, and the development of more accurate operationalisations and appropriate mathematical models, is hindered [8–10, 13, 20–25]. This limitation stifles advancements in its identification, measurement, and prediction.

**Table 1:** Core nomenclature for definitional and conceptual foundations in science

*Definitional frameworks*	
Theoretical definition	A theoretical definition is an explanation of a concept that establishes its fundamental properties and relationships, providing a conceptual framework for understanding, analysing, and distinguishing it from related concepts. The primary role of a theoretical definition is to ensure theoretical accuracy and logical consistency, capturing the fundamental essence of a concept as accurately as possible. This allows the concept to be consistently and appropriately operationalised [8, 10, 20, 21, 23, 24, 48, 57]
Operational definition	An operational definition outlines how a concept will be measured or observed in practice, specifying the procedures, criteria, or variables used to quantify and identify it within a given context [10, 20, 21, 23, 24, 48, 57, 58]. In this respect, the task of setting clear and measurable boundaries falls to the process of operationalisation
Scientific explanandum	A phenomenon or construct that is suitable for scientific explanation. To qualify as a scientific explanandum, a concept must exhibit sufficient ontological and mechanistic unity, admit principled formalisation, and allow for the specification of necessary conditions whose violation would falsify claims about its existence or behaviour [8, 10, 50, 59]. Constructs that lack clear boundaries, permit unrestricted reinterpretation, or collapse into tautology fail to qualify as scientific explananda
*Logical and philosophical foundations*	
Philosophical foundations of logic, language, science, and mathematics	A collective reference to the core philosophical disciplines that underpin scientific reasoning and conceptual analysis. The philosophy of logic concerns the principles of valid reasoning and inference; the philosophy of language examines meaning, reference, and the relation between words and concepts; the philosophy of science investigates the nature of explanation, theory formation, and empirical validation; and the philosophy of mathematics explores the abstract structures and formal systems through which scientific concepts are expressed. Together, these domains provide the conceptual and methodological groundwork for defining, analysing, and formalising scientific concepts
Aristotelian logic and essentialism (in relation to definition)	Aristotelian logic, as it pertains to definition, is a system of reasoning aimed at identifying the essence (to ti ēn einai, “what it is to be”) of a concept by distinguishing its essential properties, those both necessary and sufficient for its definition, from its accidental properties (symbebekós, “nonessential or contingent attributes”). Definitions are formed through universals (katholou, general categories) that subsume particulars (kath’ hekaston, specific instances), ensuring clarity and conceptual precision. This method classifies entities according to their substance (ousia) and associated attributes, providing a rigorous logical framework for constructing coherent and analytically robust definitions, as detailed in Aristotle’s Posterior Analytics [8]
Carnapian explication	Carnapian explication (see Rudolf Carnap’s The Methodological Character of Theoretical Concepts [12] and Logical Foundations of Probability [13]) is a method of conceptual clarification in which an imprecise, pre-scientific, or ambiguous concept (the explicandum) is refined or replaced by a more precise, logically consistent, and scientifically useful concept (the explicatum). Its aim is to enhance clarity, coherence, and applicability within a theoretical or formal system, ensuring that the concept can be rigorously defined and expressed in formally definable terms, and transformed into a logico-mathematical construct suitable for scientific use
Classical logic	Classical logic is a formal system of reasoning that originated with Aristotle’s Organon and was later refined through the development of symbolic logic. It is grounded in foundational principles such as the law of non-contradiction (a statement and its negation cannot both be true), the law of the excluded middle (every proposition must be either true or false, with no middle value), and bivalence (truth values are binary: true or false). Classical logic provides the basis for deductive reasoning, ensuring that conclusions necessarily follow from true premises, and it underpins most traditional systems of logical analysis, mathematics, and scientific inference
First principles	First principles are the most basic, foundational concepts or assumptions that cannot be deduced from any other idea. In problem-solving or reasoning, starting from first principles means breaking down complex issues into their simplest, most fundamental elements, and building understanding or solutions from these core truths. In essence, it involves repeatedly questioning until you reach the most basic truth or axiom that cannot be reduced further
Formalisation	Formalisation is the process of expressing ideas, concepts, or systems in a precise, structured, and standardised form, often using symbols, rules, and formal logic to ensure clarity and consistency
*Relevant Aristotelian concepts of definition*	
To ti ēn einai (Essence / “What it is to be”)	To ti ēn einai refers to the essential nature or defining property of a thing i.e., what it is to be that thing. It designates the set of properties that are both necessary and sufficient for the concept’s identity, distinguishing it from all others. For example, what makes a banana a banana is not its colour or size but its distinctive structure and composition, which define its nature and distinguish it from other fruits
Symbebekós (accidental property)	A symbebekós (accidental property) is a characteristic that may or may not belong to something without altering its essence. It is a contingent feature that can change while the thing itself remains what it is. For example, the colour of a banana is an accidental property. A banana can be yellow, green, or brown depending on ripeness, but a banana remains a banana regardless of its colour. In relation to injury, symptoms are accidental properties: an athlete may or may not experience pain, swelling, or any other symptom when an injury exists. Note that it is not a coincidence that both symbebekós and symptom share the Greek prefix sym- (“with”), as this denotes contingency rather than essence
Idion (proper attribute)	An idion (proper attribute) is a property that, while not part of a thing’s essence, necessarily follows from it. It is inseparable from the essence but not identical to it. For example, loss of physical function necessarily follows from tissue damage at localised levels and therefore represents an idion of injury rather than a merely accidental (symbebekós) property
*Constructs and variables in scientific representation*	
Latent construct	A latent construct is an abstract, unobservable concept (e.g., intelligence, anxiety, etc.) that cannot be directly measured. Instead, it is inferred through patterns in observable data
Latent variable	A latent variable is the statistical or mathematical representation of a latent construct, typically estimated from multiple observed indicators (also called manifest variables) in models such as factor analysis or structural equation modelling. While the construct is theoretical, the latent variable provides a measurable proxy within a quantitative framework
*Metaphysical and epistemological domains*	
Metaphysics	Metaphysics is the branch of philosophy that examines the fundamental nature of reality, exploring concepts such as existence, causality, time, and space. It also involves disentangling and clarifying concepts to provide a coherent framework for understanding the principles and structures underlying reality
Epistemology	Epistemology is the branch of philosophy that studies the nature, sources, and limits of knowledge, focusing on how we know what we know
Ontology	Ontology is the branch of philosophy that studies the nature of being, existence, and reality, focusing on the categorisation and relationships of entities and concepts
Semantics	Semantics is the study of the meaning of words, phrases, and symbols, and how they are used to convey information and concepts
*Conceptual systems and networks*	
Ontological framework	An ontological framework is a structured system that defines the fundamental categories, concepts, and relationships that exist within a particular domain of inquiry. It provides a conceptual foundation for understanding what exists, how concepts relate to each other, and the principles governing their interactions
Semantic network	A semantic network is a conceptual framework that represents relationships between concepts or entities in a structured model, facilitating understanding and inference of meaning

Currently, the International Olympic Committee (IOC) offers one of the more refined definitions of sports (athletic) injury, describing it as:

“Tissue damage or other derangement of normal physical function due to participation in sports, resulting from rapid or repetitive transfer of kinetic energy” [6] **(Definition 1)**.

This definition is widely adopted for the recording and reporting of epidemiological data on injury in sport [6], providing the theoretical framework from which various operational definitions (Table 1) of athletic injury are developed, with these typically focused on physical complaints, athlete availability for sports participation, and time-loss, i.e., time-loss injury [4, 6, 26–29]. Moreover, this definition partially aligns with broader definitions of injury (not to be confused with *athletic injury*) articulated by various authoritative sources. For instance, the World Health Organization (WHO) and International Classification of Diseases (ICD-11) define injury as:

“A bodily lesion at the organic level, resulting from acute exposure to energy (mechanical, thermal, electrical, chemical, or radiant), in amounts that exceed the threshold of physiological tolerance” [30] **(Definition 2)**.

and

“Physical or physiological bodily harm resulting from the interaction of the body with energy (mechanical, thermal, electrical, chemical, or radiant, or due to extreme pressure) in an amount, or at a rate of transfer, that exceeds physical or physiological tolerance. Injury can also result from a lack of vital elements, such as oxygen.

Poisoning by, and toxic effects of, substances are included, as is damage to or due to implanted devices” [31] **(Definition 3)**.

However, despite sharing some similarities with the definitions of injury proposed by the WHO and ICD-11, the definition of sports (athletic) injury put forward by the IOC (Definition 1) lacks conceptual coherence in some areas, particularly with respect to thresholds of tolerance, which are central to the definitions of the WHO and ICD-11.

In applied sports settings concerned with the day-to-day management of athletes, the absence of a sound theoretical definition of athletic injury may, depending on the circumstance, be of little practical significance. Here, the concept of injury is often treated as some vague amalgamation of various elements, such as tissue damage, pain, functional impairment, and psychological state, typically culminating in time away from sport. Accordingly, the exclusion of some of these components from the theoretical definition put forward by the IOC, such as pain and availability for sports participation, may appear too reductionist and confusing to some [32, 33], especially when these concepts are often implicated in various operational definitions of athletic injury [4, 6, 26–29]. However, the IOC is correct to exclude these components from their proposed theoretical definition, as including them would not only be inconsistent with the historical and current definitions of the term injury but would also conflate multiple distinct concepts that are fundamentally different from injury, i.e., they are neither necessary nor sufficient for an injury to exist [8, 9, 34]. Accordingly, including these components would constitute an error in logic that would undermine the classification and scientific process [8, 9, 12, 13, 34–40].

Despite this, in practical settings, precision of word choice and adherence to rigid definitional standards are often of secondary importance to the primary goal of efficiently conveying intended meaning [41]. For example, a coach or staff member might describe an absent athlete as “injured”, “in pain”, “unavailable to participate”, “busted”, or (insert swearword of choice), and if the intended meaning – that the athlete is unavailable to compete – is successfully conveyed (and social etiquette aside), the adopted word choice is considered effective. This highlights that words are symbols used to convey meaning [41–45], and when the focus is on shared and timely understanding rather than strict definitional accuracy, their relatively loose application is of little consequence. It follows that, in applied sporting contexts, if intended meaning is effectively communicated, individuals can adopt whichever word choices they please, and debates over specific terminology can typically be dismissed as ‘semantics,’ since the focus is on practical, context-dependent interpretation (pragmatism) [41] rather than strict technical precision and formal analysis of meaning (semantics) [8, 9, 34, 46–48].

In scientific contexts, precise language and the formal analysis of meaning take on significantly greater importance. In this context, words and their definitions play a critical role in distinguishing between concepts and phenomena so that they can be accurately identified, measured, and analysed without conflation [8–10, 34–36, 44–46, 48]. This precision is crucial for formulating hypotheses, making accurate predictions, communicating findings, and building theories that can be consistently tested and applied across contexts [8–10, 36]. Indeed, the relationship between ontology, epistemology, and semantics (Table 1) is a central component of scientific inquiry [8–11, 46], with ontology concerning itself with the nature of the entities, concepts, or phenomena to which terms refer, while semantics is responsible for defining and clarifying the meaning of those terms.

In sports science and medicine, the absence of a conceptually sound theoretical definition of athletic injury represents a significant problem, generating uncertainty about its meaning. What exactly constitutes an injury, that is, what are the boundaries of this construct? What, precisely, is being predicted? And, by extension, which processes and relationships ought to be modelled? This ambiguity impedes and obscures efforts to formalise, operationalise, and model athletic injury and associated constructs [11, 21, 23, 24], leading to logical contradictions and eroding the construct’s predictability, testability, falsifiability, and reproducibility, which are core tenets of the scientific method [10, 19, 49, 50]. Establishing a well-founded theoretical definition of athletic injury that appropriately captures its fundamental essence is therefore crucial, as it provides the foundational framework upon which appropriate operational definitions are developed, facilitating advancements in the identification, measurement, mathematisation, and prediction of athletic injuries, and a clearer understanding of any limitations inherent in any chosen operational criteria.

**Fig. 1:**
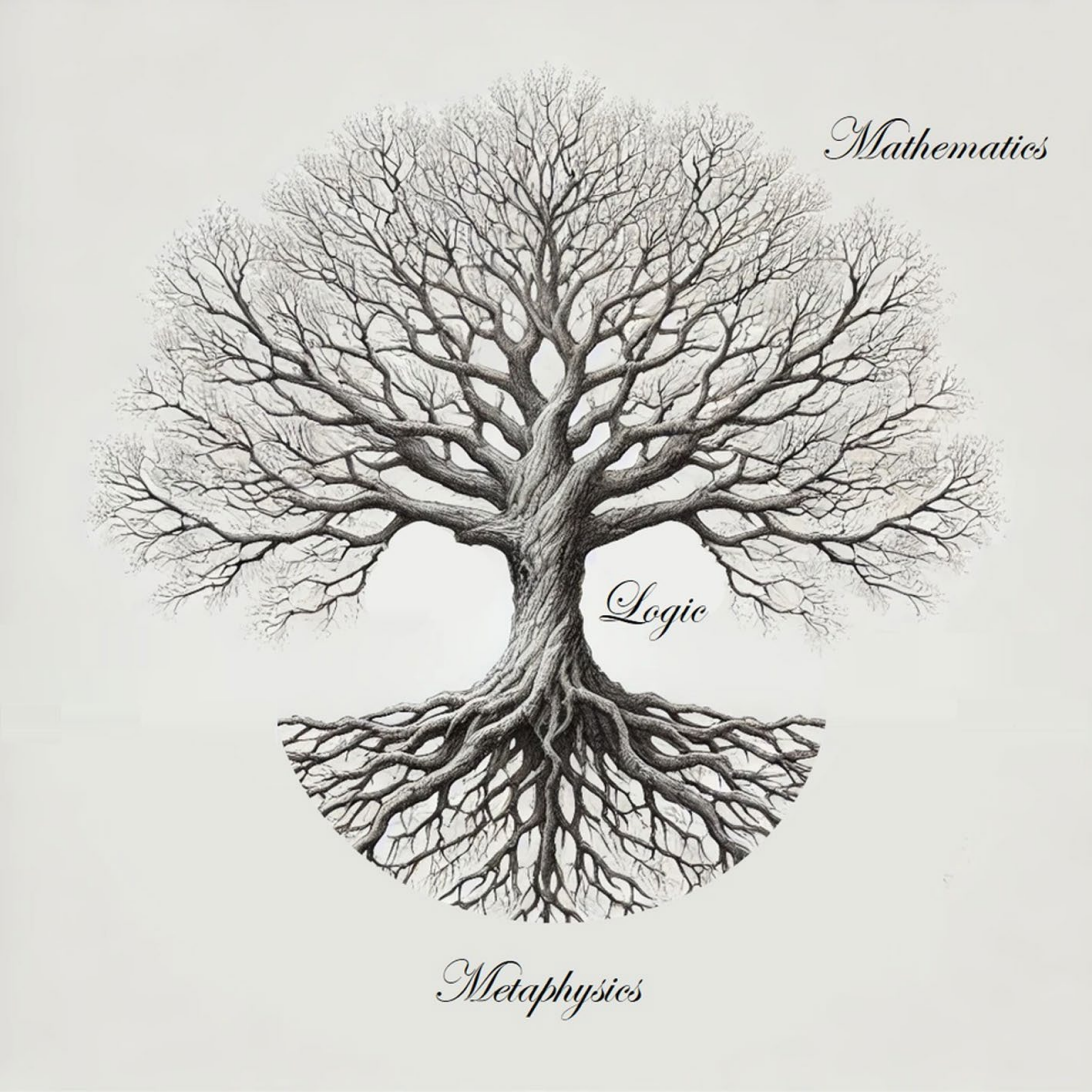
The Tree of Precision. Inspired by and adapted from Descartes’ *Tree of Philosophy*, which encompasses metaphysics, physics, and the other sciences (medicine, mechanics, and morals) [55], the *Tree of Precision* illustrates the hierarchical process of refining, formalising, and mathematising scientific concepts adopted in this article. The roots symbolise metaphysical inquiry, providing the foundational clarity necessary for disentangling and defining concepts [8, 10, 11, 39, 40, 53]. The trunk represents logical reasoning, ensuring structural coherence and logical consistency – a prerequisite for the development of formal and mathematical systems [10, 12, 13, 22, 35]. The branches and leaves embody mathematics, the universal language of precision, through which clarified concepts are operationalised into quantifiable and predictive frameworks [11–13, 23–25, 54, 56]. For an insightful discussion on the desirability of formalisation in science, see Suppes [23]

Given these considerations, the aim of this article is ambitious: to develop a robust, conceptually and logically coherent theoretical definition of athletic injury that effectively captures its fundamental essence (Aristotle’s *to ti ēn einai, “*what it is to be”; Table 1 [8]). The approach adopted to achieve this (illustrated in Fig. 1) is informed by the philosophies of logic, language, science, and mathematics (Table 1) and employs a structured process of metaphysical analysis and Carnapian explication [12, 13], grounded in Aristotelian essentialism, classical logic, and reasoning towards first principles (Table 1) [8, 39, 40]. Specifically, this process employs a series of thought experiments, boundary tests, and logical arguments to identify the core attributes that are essential to defining athletic injury. These tools are designed to test the logical boundaries distinguishing athletic injury from non-injury and other related phenomena. They disentangle conflated concepts (i.e., Aristotle’s *symbebekós*, ‘accidental properties’; Table 1 [8]), resolve existing logical inconsistencies, and establish a set of necessary and sufficient conditions (i.e., essential properties) needed for an athletic injury to exist [8, 9, 34, 35, 37, 40, 47, 51–53].

Once conflated concepts have been tested for logical independence and successfully disentangled, and athletic injury has been theoretically defined in a coherent manner, the critical precondition for formalisation is established. Building on this foundation, the construct of athletic injury is then formalised as a logico-mathematical entity for application in mathematical modelling (e.g., predictive modelling, simulation, and causal inference). This, in turn, provides the basis for a formalised ontological framework and semantic network (Table 1) surrounding athletic injury, offering a structured mathematical system that seamlessly integrates related constructs such as severity, recovery, and rate of recovery [22, 23, 54].

Ultimately, the procedures outlined in this article clarify and align the theoretical, observational, and mathematical dimensions of athletic injury and anchor it, together with its associated constructs, in objectively measurable physical parameters that can be meaningfully integrated into probabilistic models. Through this process, athletic injury and its related constructs are transformed from vague notions, subject to inconsistent interpretations and applications (i.e., bias), into coherent logico-mathematical objects with precisely defined relationships [23, 24, 54], thereby enhancing the empirical testability and reproducibility of athletic injury research [10, 23].

## A Brief Introduction to Logical Reasoning, Necessity and Sufficiency, Thought Experiments, and Boundary Testing

*“If what is seen and experienced is portrayed in the language of logic, we are engaged in science. If it is communicated through forms whose connections are not accessible to the conscious mind but are recognized intuitively as meaningful, then we are engaged in art.”* – Albert Einstein [60]

Logical reasoning is a cornerstone of both metaphysical and scientific inquiry, enabling researchers to construct valid arguments (Table 2), evaluate concepts and definitions, and systematically interpret evidence [8, 10, 22, 35, 36, 39, 45, 53, 61–65]. Through logical reasoning, arguments can be assessed for soundness and consistency (Table 2), flaws in reasoning can be identified, and coherent frameworks for understanding complex phenomena developed [7, 8, 10, 35, 39, 45, 61, 65–67]. This structured approach typically involves applying deductive and inductive methods (Table 2) to distinguish valid arguments from invalid ones [8, 10, 36, 39, 53, 61, 68, 69], ensuring that conclusions are derived from objective, logically consistent criteria rather than subjective biases [8, 10, 35, 36, 39, 40, 45, 53, 61, 62, 65, 68, 69].

**Table 2:** Relevant nomenclature for logical reasoning and analytical tools

*Forms of reasoning*	
Logical reasoning	Logical reasoning is the process of using structured, coherent thinking to analyse information, draw conclusions, and solve problems based on principles of validity and soundness. It involves identifying relationships between concepts, evaluating evidence, and applying rules of logic to reach conclusions that are consistent with given premises
Deductive reasoning (Deduction)	Deduction is a form of reasoning where specific conclusions are logically derived from general premises. If the premises are true, the conclusion must also be true. Such reasoning often takes the form of a syllogism
Inductive reasoning (Induction)	Induction is a form of reasoning where general conclusions are drawn from specific observations. The conclusions are probable but not guaranteed to be true
*Logical relations and conditions*	
Necessary condition	A necessary condition refers to a condition or requirement that must be true or satisfied for a particular statement, outcome, or event to occur
Sufficient condition	A sufficient condition refers to a condition that, if met, guarantees a particular outcome or event
*Analytical and conceptual testing methods*	
Argument	An argument is a structured set of premises offered to support or refute a conclusion, with the goal of showing whether the conclusion logically follows from the premises. Arguments fall into three possible categories: valid and sound, valid and unsound, and invalid and unsound. A fourth category, sound but invalid, is impossible because soundness entails validity. When assessing whether a definition is conceptually superior, the supporting argument must fall into the valid-and-sound category
Thought experiments	A thought experiment is a mental exercise used to explore various scenarios (e.g., factual scenarios, counterfactuals, hypotheticals, etc.), analyse concepts, test logical boundaries, or evaluate the implications of ideas without the need for physical experimentation. By constructing and manipulating these scenarios, and applying deductive and inductive reasoning methods, thought experiments help reveal logical inconsistencies and consequences and provide insights into complex problems or theories
Boundary testing	Boundary testing is a process of evaluating the limits of a system, concept, or theory by examining how it behaves or holds true at the extreme edges of its defined parameters
*Logical structures and rules of inference*	
Validity (in logic)	Validity refers to the logical structure of an argument. An argument is valid if, assuming the premises are true, the conclusion must also be true. Validity concerns the form of reasoning, not the truth of the premises themselves
Tautology	A tautology is a statement or proposition that is true by virtue of its logical form alone, regardless of the content of its terms (e.g., “If it is raining, then it is raining”). In logic, tautologies are analytically true and cannot be false. While they are important for ensuring internal consistency within formal systems, when used in conceptual or definitional contexts, tautologies indicate circularity or vacuity, i.e., statements that provide no new information or empirical content. In scientific reasoning, such tautological definitions are problematic because they render a construct unfalsifiable and empirically meaningless, collapsing into circular statements such as “anything that is identified as an injury is an injury”
Soundness	Soundness refers to both the truth of an argument’s premises and the validity of its structure. An argument is sound if it is valid and all its premises are true. Therefore, every sound argument is valid, but not every valid argument is sound
Consistency	Consistency refers to the absence of contradiction within a set of statements or propositions. A consistent argument or system does not contain both a statement and its negation as true at the same time. Consistency is essential for maintaining logical coherence and reliability in reasoning
Syllogism	A syllogism, developed by Aristotle in his Prior Analytics [38], is a form of deductive reasoning consisting of a major premise, a minor premise, and a conclusion that follows logically from the premises. It establishes valid relationships between concepts, ensuring that if the premises are true, the conclusion must also be true
Refutation by counterexample	Refutation by counterexample is a method of disproving a general claim or universal statement by presenting a single example that contradicts it. Because a universal claim asserts that something is true in all cases, one valid counterexample is sufficient to demonstrate that the claim is false, consistent with the logic of modus tollens
Modus tollens	(Latin) The rule of logic which states that if a conditional statement (‘if p then q’) is accepted, and the consequent does not hold (not-q) then the negation of the antecedent (not-p) can be inferred
Reductio ad absurdum	(Latin: “reduction to absurdity”) In logic, reductio ad absurdum is a form of refutation showing contradictory or absurd consequences following upon premises as a matter of logical necessity
Ex contradictione quodlibet (the law of explosion)	(Latin: “from contradiction, anything follows”) In classical logic, this is the principle that once a contradiction is present in a system, that is, both a statement and its negation are held to be true, any proposition whatsoever can be logically derived. This leads to what is known as logical explosion, where the presence of inconsistency undermines the reliability of the entire system. The principle underscores the necessity of consistency in formal logic
*Philosophical orientation in logic*	
Logical positivists	Logical positivists, a group of twentieth-century philosophers associated with the Vienna Circle, argued that meaningful statements must be empirically verifiable or analytically true. They rejected metaphysical, ethical, and theological claims as meaningless if they could not be tested through observation or logic. Emphasising science and formal reasoning, they sought to distinguish scientific knowledge from unverifiable assertions

To better introduce these, an explanation with some examples of deductive and inductive reasoning may prove useful. Deductive reasoning starts with general principles or premises and applies them to specific cases, establishing conclusions that are logically certain if the premises are true [39, 61]. For example, consider the following syllogism (Table 2):

All 100 m Olympic gold medallists are human.

Usain Bolt is a 100 m Olympic gold medallist.

_______________________________

Therefore, Usain Bolt is human.

This type of inference is deductive because given that the premises are true and the reasoning is valid, then the conclusion must be true. It is absolute in its logic. Deductive reasoning is particularly useful for defining necessary conditions, highlighting what conditions are needed for a concept or definition to hold [8, 9, 34, 36].

Inductive reasoning, on the other hand, involves drawing general conclusions from specific observations. For instance, consider the following premise and conclusion:

Jamaicans have performed well in the past in the 100 m event at the Olympics.

________________________________________

Therefore, Jamaicans will perform well in future 100 m events at the Olympics.

Of course, the presented inference is probabilistic and not a certainty. It is for this reason that, depending on the context, deduction may be preferable to induction in the philosophy of science, allowing for logically certain conclusions (assuming the premises are valid) [8, 53, 61, 64, 70, 71]. However, while inductive reasoning does not guarantee certainty, it is fundamental to scientific inquiry, forming the basis of observation, experimentation, and empirical generalisation [10, 36, 50]. Through induction, patterns and relationships can be identified, guiding both the development of hypotheses and the interpretation of experimental results [10, 19, 36, 50].

By integrating deductive and inductive approaches, researchers can develop more comprehensive theoretical models and precise definitions. These methods enable the use of thought experiments, boundary tests, and logical arguments to eliminate logical inconsistencies, disentangle and refine concepts of interest, and identify necessary and sufficient conditions for a concept to be upheld [8, 34, 39, 61, 72]. Through these methods, logical reasoning contributes to a deeper understanding of the underlying principles that define a theory or concept [8, 10, 34, 35, 39, 45, 61, 62, 65].

### Necessity and Sufficiency

Necessity and sufficiency (Table 2) are foundational criteria in the philosophies of logic, language, science, and mathematics for constructing precise theories and definitions [8–10, 34–37, 39, 40, 48, 53, 55, 63, 65, 72–75]. These criteria determine which conditions must be met (necessary) and which are enough (sufficient) to define the boundaries of a concept [8, 9, 34]. Consequently, they play a crucial role in forming definitions by clarifying core attributes and eliminating logical inconsistencies or conflated ideas, ultimately leading to a more systematic and coherent understanding of various phenomena and concepts [8, 9, 34].

To elaborate, a necessary condition is one that must be met for a concept to apply. This allows for deductive inferences as if the condition is not satisfied, the concept or phenomenon cannot hold. For example, consider the following scenario:

Being human is a *necessary condition* for competing in the 100 m at the Olympics (as per current and historical rules).

Usain Bolt competes in the 100 m at the Olympics.

____________________________________

Therefore, Usain Bolt is human.

Here, if the necessary condition is accepted as valid (and setting aside, for the sake of this example, the fact that Usain Bolt is now retired), it facilitates deductive reasoning, as either the condition is met and the concept is upheld, or it is not. However, while being human is a necessary condition for competing in the 100 m at the Olympics, it is not a *sufficient condition*, as not all humans compete in this event. Indeed, a sufficient condition is one that, when met, guarantees that the concept or definition applies [8, 34, 36, 67, 76]. For example, consider the following scenario:

Winning the 100 m final at the Olympics guarantees a gold medal.

________________________________

Therefore, winning the 100 m final is a *sufficient condition* for being an Olympic gold medallist.

Here, winning the 100 m final at the Olympics is considered a sufficient condition for being an Olympic gold medallist because it satisfies all criteria needed for this classification. However, there may be more than one sufficient condition, as is the case with winning an Olympic gold medal. Being an Olympic gold medallist can result from winning events other than the 100 m final, such as the javelin or high jump events.

When developing theoretical definitions, such as a fundamental theoretical definition for athletic injury, it is important to identify both necessary and sufficient conditions that define a concept to achieve conceptual clarity. This process refines definitions by distinguishing essential features (*to ti ēn einai*) from those that are merely associated (*symbebekós*), removing logical inconsistencies and reducing vagueness, resulting in a more precise and reliable understanding of a concept [8, 10, 34, 39, 40].

### Thought Experiments and Boundary Testing

The process of refining theoretical definitions through logical reasoning often incorporates the application of “boundary tests”. Boundary testing involves pushing a definition to its conceptual limits, typically through a series of thought experiments (Table 2) [8, 14, 34, 40, 72, 77–80]. While many of these thought experiments may appear extreme in nature, pressing at the edges of a concept, to dismiss them as such is to misunderstand their purpose, as this is a fundamental strength. By ‘testing (logical) boundaries’, these experiments explicitly highlight potential logical inconsistencies or cases where any proposed necessary or sufficient conditions break down, and the concept or definition does not hold. Accordingly, thought experiments have an important role in refining definitions by testing for logical independence, disentangling conflated concepts, and more clearly defining the boundaries of various concepts of interest. In science, defining a concept’s boundaries is critical for its appropriate conceptualisation, operationalisation, and investigation using the scientific method [8–12, 23, 24, 36]. This facilitates testability and consistency across studies, enabling valid comparisons and more reliable conclusions [8–11, 23, 24, 36].

## Developing a Fundamental Theoretical Definition of Athletic Injury

*“By the procedure of explication we mean the transformation of an inexact, prescientific concept, the explicandum, into a new exact concept, the explicatum… The explicatum must be given by explicit rules for its use, for example, by a definition which incorporates it into a well-constructed system of scientific either logicomathematical or empirical concepts”* – Rudolf Carnap [13]

Considering the IOC is a leading authority in global sports and its definitions significantly influence international standards and practices [6], to provide a starting point for developing a fundamental theoretical definition of athletic injury, there is arguably no better place to begin than to revisit the current definition proposed by this organisation (Definition 1) [6]. Here, there are a series of key features that highlight identified necessary conditions for an athletic injury to exist, as proposed by the IOC: (1) tissue damage or other derangement of normal physical function, (2) due to participation in sports, and (3) resulting from rapid or repetitive transfer of kinetic energy. Some of these conditions may be surprising to some. Why does tissue damage or other derangement of normal physical function warrant inclusion, but pain does not? Is this not an important component of athletic injury worthy of inclusion? What about other symptoms such as swelling and tissue inflammation? Or availability for sports participation? Certainly, various operational definitions of athletic injury have incorporated or imply many of these elements [4, 6, 26–29]. For example, in soccer, Fuller et al. [29] operationally defined injury as:

“Any physical complaint requiring medical attention resulting in a missed A-League match” [29] **(Definition 4)**.

Similarly, Ekstrand et al. [26, 29] have operationally defined injury as:

“Any physical complaint sustained by a player that resulted from a football match or football training and led to the player being unable to take full part in future football training or match play” [26, 29] **(Definition 5)**.

So why then, would symptoms such as pain and swelling, which are implied in “any physical complaint”, or athlete availability for sports participation, be excluded from a theoretical definition of athletic injury? Are these not necessary or sufficient conditions for an athletic injury to exist? The following sections provide a logical examination of the conditions proposed within the theoretical definition put forward by the IOC, as well as the absence of those conditions for which exclusion may appear confusing to some.

### Disentangling Conflated Concepts: T*o ti ēn einai* (Essence) versus *Symbebekós* (Accidental Properties)

*“The philosopher’s task consists principally of disentangling our concepts. It does not aim so much at arriving at new truths as it (does) coming to understand better those which we have already arrived”* – Michael Dummett [1]

#### The Exclusion of Pain and Other Symptoms

Considering pain and other symptoms such as swelling and inflammation are important considerations in the practical management and diagnosis of athletic injury in applied sports settings, as well as large-scale epidemiological studies, their absence from the proposed theoretical definition from the IOC may appear counterintuitive. Indeed, these concepts are commonly conflated with injury [32, 33]. However, their exclusion is logically accurate.

To illustrate this, consider the following thought experiment: An athlete breaks their leg during a soccer match after a poorly timed slide tackle from the opposition. Reasonably, the athlete has sustained an injury; their leg is broken, they are in excruciating pain, and must be stretchered from the field. To assist with this pain, the doctors administer an anaesthetic, and shortly after, the athlete no longer experiences pain. Is the athlete still considered to have an injury despite the absence of pain? To answer no would be unreasonable as per the common definitions and uses of the term [6, 30, 31, 81–84]. The athlete’s leg is broken, and they are surely unable to play for an extended period, facing extensive surgery and months of recovery to mend their broken leg. It would be a strange claim indeed if one was to state that the anaesthetic removed the injury.

While this thought experiment may appear ‘extreme’ to some, injuries in sports that require painkillers, anaesthetics, and surgical intervention, for example, anterior cruciate ligament (ACL) ruptures, Achilles tendon ruptures, bone fractures, etc. are not uncommon, while other instances exist where physical injuries are clearly present in humans, but pain does not present or subsides for various reasons, as seen in traumatic military injuries [85–87]. More importantly, however, any perceived extremity of this thought experiment is ultimately irrelevant, as it serves a clear and concise logical purpose. From this scenario, an important conclusion can be deduced from the following premises:

The athlete has an athletic injury.

The athlete is not experiencing pain.

______________________________

Therefore, pain is not a *necessary condition* for an athletic injury to exist.

By presenting even a single scenario where an athletic injury exists but pain does not, and adhering to the logical principles of *refutation by counterexample* and *modus tollens* (Table 2), any claims that pain is a defining feature of athletic injury are falsified. This approach mirrors the classical example of falsification in science: to falsify the claim that all swans are white, observing a single black swan is sufficient, regardless of how many white swans have been observed previously [10]. As a result, assertions that pain is a necessary condition or defining feature of athletic injury are logically refuted and rendered untenable. Any perceived extremity of the proposed hypothetical, or the inclusion of an exogenous substance (such as an anaesthetic) to remove the pain, is of no relevance. In fact, this reflects the fundamental strength of the thought experiment: a single counterexample (*refutation by counterexample* [8, 10, 22, 34, 39, 75]) is sufficient to falsify a universal claim (*Katholou* [8]), providing a clear demonstration of absolute logic where the conclusion that pain is not a necessary condition for athletic injury deductively follows from the premises [8, 34].

However, this thought experiment does not end here, as it is still possible that pain is a sufficient condition for an athletic injury to exist. To address this, let us consider other scenarios where tissue damage does not exist, but pain does. Is it reasonable to consider these scenarios as athletic injuries? No, it is not. Pain may arise during sports participation for many reasons unrelated to athletic injury, for example, distinct medical conditions such as angina, cancer, blood clots, autoimmune diseases, and neurological disorders can all lead to pain when participating in sports. Labelling such conditions as athletic injuries would result in an unreasonably broad application of the term, effectively categorising all medical conditions involving pain as injuries. Accordingly, the following premises can be set:

The athlete is experiencing pain due to a medical condition, such as an autoimmune or neurological disorder.

The athlete has not sustained an athletic injury.

Therefore, pain is not a *sufficient condition* for an athletic injury to exist.

Through the presented thought experiments, it can be conclusively deduced that athletic injury and pain are distinct concepts, and pain has no place in a fundamental theoretical definition of athletic injury. By adhering to the principles of necessity and sufficiency, any claim that pain is a defining feature of injury can be rejected via a *reductio ad absurdum* (Table 2), a formal logical procedure which demonstrates the falsity of an assumption by showing that it leads to absurd or contradictory outcomes. In short, since pain is neither necessary nor sufficient for an athletic injury to exist, this directly contradicts any initial assumption that pain is a defining feature of injury, reducing it to absurdity. Accordingly, it is clear that including pain within a fundamental theoretical definition of athlete injury would be logically unsound [8], conflating two associated but separate phenomena, whereby the second phenomenon is a contingent or ‘accidental’ property (*symbebekós*) of the first [8, 34]. On this basis, the IOC’s decision to exclude pain from its proposed definition of injury is logically justified and theoretically sound.

#### Implications for the Scientific Method: From Vagueness to Scientific Incoherence

*“A theory which is not refutable by any conceivable event is non-scientific. Irrefutability is not a virtue of a theory (as people often think) but a vice… Every genuine test of a theory is an attempt to falsify it, or to refute it. Testability is falsifiability; but there are degrees of testability: some theories are more testable, more exposed to refutation, than others; they take, as it were, greater risks”* – Karl Popper [19]

Thought experiments and logical analysis, such as those presented here, are not merely abstract philosophical exercises. Rather, they reveal foundational issues that directly affect scientific inquiry. Erroneously including pain as a defining feature of athletic injury, when pain is neither necessary nor sufficient for an injury to exist, has serious implications for the scientific study of this concept. It introduces logical incoherence, leads to fundamental errors of categorisation, and undermines efforts to develop testable, predictive, and falsifiable models (Table 3).

**Table 3:** Principles of the scientific method and validation

*Definitional frameworks*	
Predictability	Here, predictability refers to a theory’s ability to generate specific, testable predictions about future observations or experiments. It implies that the theory should outline what outcomes are expected under certain conditions and what results would contradict the theory. Predictability is crucial for falsifiability, as it establishes clear criteria for testing and determining whether the theory can be refuted, thereby making it scientifically meaningful. Without predictability, a theory cannot be tested and, thus, cannot be falsified [10]
Testability	Testability refers to the degree to which a concept, theory, or hypothesis can be empirically examined through observation, measurement, or experimentation. It bridges the theoretical and operational levels of science by ensuring that abstract concepts can be linked to observable phenomena through measurable indicators or correspondence rules
Falsifiability	Falsifiability is a fundamental criterion in the scientific method, referring to the degree to which a hypothesis or theory can be shown to be false through observation or experimentation. A falsifiable theory must make precise, testable predictions that may be contradicted by empirical evidence. As philosopher Karl Popper argued, if a theory cannot be tested or potentially refuted in this way, it does not qualify as scientifically valid and instead begins to fall into the realm of pseudoscience. This is because a theory that cannot be invalidated is immune to critical evaluation [10] It is important to note that this principle is not without some controversy. Falsifiability is often seen as an ideal of precision rather than an absolute criterion, as real-world scientific testing can never yield deductive (definitive) refutation. Instead, theories are generally subjected to repeated testing, and scientists aim for theories with a *high degree of falsifiability*, continually refining them as new evidence emerges
Reproducibility	Reproducibility is the extent to which consistent results can be obtained using the same methods, data, and conditions when an experiment or study is repeated by different researchers or at different times

To illustrate, consider a scenario in which injury is ‘defined’ to include both tissue damage and pain (and additional concepts if desired), yet neither, individually nor in combination, is necessary or sufficient for an injury to exist. Under such conditions, the construct of injury becomes vacuous, i.e., empirically underdetermined [9–11, 21, 88, 89]. Indeed, such an interpretation of injury accommodates all possible combinations of tissue damage and pain (whether present, absent, or discordant), thereby eliminating any formalised criteria for determining whether an injury does or does not exist. Consequently, these so-called ‘defining properties’ of injury become functionally redundant, contributing nothing to the construct’s operationalisation.

The consequence of this incoherence is that injury is functionally reduced, in operational practice, to a subjective mental experience: an injury exists simply because someone says that it does (*but what are they claiming exists?)*, and no empirical evidence can verify or falsify the claim [9–11, 21, 88, 89]. The construct becomes tautological (Table 2), self-referential, and unfalsifiable [10]. An athlete can fake having an injury because they want a holiday, and no empirical data can dispute the injury claim. Indeed, how can one ‘fake’ having an injury if the construct itself permits no falsifiable boundary and the only criterion is self-identification? This same logic holds for the injury claims of practitioners. One practitioner can assert that an athlete has an injury, while another can assert that they do not, and either position remains beyond empirical refutation. The result is a declaration without reference, and a concept without content, that is immune to empirical scrutiny. Some philosophers of science, such as logical positivists (Table 2), would likely argue that such definitional vagueness renders statements like *“this athlete has (or has not) sustained an injury”* empirically meaningless, as it would lack clearly specifiable observational content [9, 11, 88, 89]. From this perspective, a construct that cannot be anchored to observable indicators may not belong in scientific discourse at all and requires reconstruction with measurable criteria or exclusion from empirical inquiry altogether [9, 11, 88, 89].

Vacuous constructs of this kind have been extensively critiqued in the scientific literature for undermining the integrity of the scientific method. Paul Meehl, who helped establish the modern framework for construct validity [20], cautioned against such conceptually empty terms on the grounds that they lack substantive theoretical and empirical content [21]. Similarly, within Karl Popper’s philosophy of science, a construct that lacks falsifiability and permits unlimited reinterpretation of evidence falls outside the domain of scientific explanations and meets Popper’s criteria for pseudoscience [19]. This approach also reflects Popper's broader critique of psychologism, in which objective knowledge claims are improperly reduced to subjective mental experiences, undermining epistemological objectivity [10]. In such cases, theories explaining injury can be constructed post hoc and remain insulated from empirical contradiction [10, 19].

While these criteria are not absolute given that theories are never empirically verifiable or falsifiable in an absolute sense [10, 61], they nonetheless highlight a critical issue for scientific inquiry: the absence of clearly specified criteria by which injury can be reliably identified, falsified, or predicted across contexts and cases. This lack of definitional clarity obstructs the development of predictive, testable, and falsifiable theories, which is an issue at the heart of Popper’s demarcation problem [10, 19]. It also introduces interpretive bias, undermines reproducibility (Table 3), and erodes the concept’s reliability and scientific utility [2, 8, 10, 11, 23, 34].

Ultimately, the same logical concerns highlighted here would hold for all other symptoms of injury such as swelling or bruising, which may or may not accompany an injury. It is for such reasons that these conditions are commonly termed *symptoms* of injury, as by definition, *symptoms* indicate but do not define a condition [90]; they are *symbebekós* [8] (note the shared Greek prefix *sym*, meaning “with”, denoting contingency rather than essence). To include them in the fundamental theoretical definition of athletic injury would be to mistakenly conflate contingent properties and observable correlates with the condition itself (*to ti ēn einai*) [8, 34].

Importantly, the distinction between pain and injury does not diminish the significance of pain, swelling or any other symptoms of injury in the practical management of athletes. Symptom assessments provide timely and cost-effective indicators (correlates) of injury that offer value for their practical assessment, management, and rehabilitation. Furthermore, symptoms (such as pain) may, depending on the context, be of more clinical concern than the actual underlying physical injury. The purpose of disentangling pain and other symptoms from the concept of athletic injury was to highlight that: (1) each of these (injury, pain, swelling, etc.) represent distinct yet associated constructs, each worthy of independent consideration and scientific investigation, and whose interrelationships can be explored and modelled using the scientific method; (2) while common symptoms may offer practical value, they arise a posteriori and are therefore unsuitable for predicting injury prior to its occurrence. Furthermore, they are also ultimately limited and unreliable as definitive measures of injury [85, 91–94], underscoring the importance of more precise, objective, and mathematically formalised frameworks; (3) applied practitioners are faced with the difficult task of managing a variety of phenomena beyond simply injury; and (4) for the purposes of precision and prediction within the sciences, it is critical that distinct phenomena (such as pain and injury) are conceptually disentangled so that advancements in operationalisation, identification, measurement, and prediction may be achieved [10, 21, 23, 24].

#### Athlete Availability as a Boundary of Demarcation?

To provide a practical demarcation boundary to assist with distinguishing athletic injury from non-injury in applied sports settings, and in particular large-scale epidemiological studies, operationalisations of athletic injury (typically of the theoretical definition proposed by the IOC; Definition 1) have commonly centred around athlete availability for sports participation and time-loss, i.e., whether an athlete is available to participate in training or match play, for example, Definitions 4 and 5 [26, 28, 29]. Accordingly, the exclusion of athlete availability from the definition of injury proposed by the IOC may be confusing to some. Is a discontinuation of sports participation neither a necessary nor sufficient condition for an athletic injury to exist?

Operational definitions are essential for translating theoretical concepts into empirically assessable constructs and measurable variables, enabling researchers to identify, measure, and evaluate them in ways that support scientific prediction and testing [10, 11, 20, 21, 48, 57, 58]. However, these definitions often sacrifice theoretical rigour to accommodate the limitations of available assessment tools, prioritising practicality (e.g., cost, feasibility, and technological constraints) over conceptual precision. Depending on the context, this can be problematic [95, 96]. As operationalisations deviate further from the constructs they are intended to represent, identification and measurement quality declines, and the link between theory and empirical science weakens, eroding explanatory power and predictive accuracy [10, 11, 20, 24].

While availability for sports participation serves as a practical criterion for demarcating injury from non-injury in applied settings and large-scale epidemiological studies, arguably aligning more closely with what sporting organisations often prioritise, which is whether an athlete is available to train or compete, it is important to recognise that defining athletic injuries in this manner constitutes a major theoretical compromise. This is why such an approach is commonly termed a ‘time-loss injury’, which is a different concept to an ‘athletic injury’.

Regardless, the concept of time-loss is fundamentally grounded in an absence of participation, and accordingly, it is important to examine whether availability for participation in sports, and by extent ‘time-loss’, is a necessary or sufficient condition for an athletic injury to exist.

Consider the following scenario: in 2008, Tiger Woods won the US Open in golf despite competing with a torn ACL and a double stress fracture in his leg. Based on any reasonable interpretation of the term injury, it would be illogical to suggest that Tiger Woods did not have an athletic injury – his ACL was ruptured, and he required knee reconstruction surgery after the tournament. Therefore, the following premises can be established, leading to a deductive conclusion:

Tiger Woods has an athletic injury.

Tiger Woods is participating in sport despite having an athletic injury.

___________________________________

Therefore, an absence of sports participation is not a *necessary condition* for an athletic injury to exist.

Tiger Woods is far from the only example of perseverance through injury to achieve a sporting goal. Just as Diomedes continued to fight after being struck by an arrow during the Battle of Troy, the sporting world is filled with heroic examples of athletes enduring injuries in their quest for sporting glory. Accordingly, it is clear that an absence of sports participation is not a necessary condition for an athletic injury to exist. However, perhaps it is sufficient? No, it is not. There are many reasons an athlete may make themselves unavailable for participation. Perhaps they are angry at their team for not passing them the ball and no longer want to play, or perhaps they are faking an injury because they are hungover. Perhaps they simply want to go on holiday for a few weeks. It would be absurd to classify such situations as injuries. Hence, availability to participate in sport is neither a necessary nor sufficient condition for an athletic injury to exist, and the IOC is correct to exclude it from their theoretical definition of injury.

#### Ex Contradictione Quodlibet (The Law of Explosion): From Contradiction, Anything Follows

Athletic injury cannot be coherently defined both as a condition determined by an absence from sporting participation and as a state that allows continued participation. Athletes often compete while injured, as in Tiger Woods’ case, which contradicts a definition based on unavailability and time-loss. In science and formal logic, contradiction is no trivial matter: by the principle of *Ex Contradictione Quodlibet* (Table 2), otherwise known as *the law of explosion*, the presence of a contradiction renders a concept or system incoherent, allowing any proposition to follow, no matter how absurd. It follows that definitions in violation of the law of non-contradiction undermine both theoretical clarity and empirical testability.

Athlete availability and injury are distinct but associated concepts, with time-loss serving as a *symbebekós* of injury. Athlete availability is not a theoretical determinant of injury, and the choice to abstain from sports participation is merely an a posteriori correlate of injury that can be shaped by an almost infinite array of extraneous factors such as pain tolerance, the intensity of competition, intrinsic motivation, or even more transient issues such as faking an injury, a hangover, or a spat with the coach. While operationalising injury as an absence from sports participation offers practical utility for tracking and management, for the purposes of precision, definition, and prediction within the sciences, it remains a necessary a priori requirement to anchor ‘time-loss injury’ to the underlying tissue damage state prior to its existence. Otherwise, there is no principled basis for distinguishing an injury from withdrawal for non-injurious reasons, undermining empirical tractability.

These concerns do not preclude the use of such correlates (e.g., time-loss, complaints, and similar measures) in contexts such as large-scale epidemiological studies, as they can still provide practical utility. However, it does emphasise that they must not be mistaken for essential definitions of injury. These constitute weak and imprecise operationalisations that prioritise convenience over conceptual precision and predictive validity, constituting a major theoretical compromise that diverges from the fundamental concept. In doing so, they promote a form of psychologism misaligned with the physical nature of injury and undermine the precision required for valid, reproducible scientific inference and predictive modelling [10, 19]. Indeed, compromises such as these can distort research findings [95, 96], conflating athletic injury with subjective decisions around availability rather than objectively assessing the injury.

To address such issues, some studies incorporate additional measures, such as MRI or other imaging techniques, to confirm the presence of underlying physical damage [97–99]. While these approaches certainly offer a superior level of precision, it is also important to recognise that the absence of identifiable lesions does not necessitate an absence of damage; it may also reflect limitations in identification and measurement technologies or processes (e.g., damage exists but observable lesions are yet to form, imaging resolution, radiographer expertise, etc.). Accordingly, advancements in damage and injury assessment technologies (e.g., higher-resolution imaging), analysis methodologies (e.g., artificial intelligence analysis, data integration, and mathematical modelling), and greater accessibility to such tools may, in time, drive further progress in this field, enabling more precise and consistent reporting of athletic injuries while disentangling them from subjective decisions surrounding athlete availability and pain tolerance.

### Tissue Damage and Transfer of Energy: The Necessary Essence (*to ti ēn einai*) of Injury

*“A definition which gives the real nature of a thing also gives its cause, and thus differs only in form from demonstration”* – Aristotle [100]

Considering pain, swelling, athlete availability and other associated concepts are neither necessary nor sufficient for an athletic injury to exist, it may appear confusing to some that tissue damage and the transfer of kinetic energy warrants inclusion. Are these not subject to the same arguments? Simply, the answer is no, although the transfer of kinetic energy alone provides an incomplete explanation, a point that will be elaborated on shortly. One explanation lies within the manner through which athletic injuries are formed, and an important necessary causal condition that underpins this. Let us revisit the definition presented by the IOC (Definition 1), whereby the following necessary conditions are presented: (1) due to participation in sports, and (2) resulting from rapid or repetitive transfer of kinetic energy. Together, these two conditions play an important role in defining the boundaries of this concept and distinguishing sports or athletic injury from general injury [30, 31, 84, 96, 101–104].

The first condition presented here is hardly controversial, as it is only reasonable that for a sport or athletic injury to exist, it must have occurred during participation in sports or athletic activities. This distinguishes these injuries from injuries that occur in other contexts outside of sport, such as workplace or household accidents. However, the second condition is of notable significance, providing a bold causal condition that an athletic injury results from rapid or repetitive transfer of kinetic energy, i.e., a transfer of kinetic energy is a necessary cause of athletic injury. This condition is partially reflected in other descriptions of athletic injury presented in the literature, which describe an athletic injury as occurring when the stresses and strains experienced by a tissue result in damage severe enough to be considered an injury [105, 106]. Note that the area under a stress–strain curve represents the energy absorbed during tissue deformation, which is sometimes (but not exclusively) due to a transfer of kinetic energy.

Given that the transfer of kinetic energy has been identified as a necessary cause for an athletic injury to occur, it is important to evaluate whether this condition is logically sound. Consider a range of some of the most common and significant injuries in sport, such as ACL ruptures, leg fractures, hamstring tears, Achilles tendon ruptures, concussions, and shoulder dislocations. These injuries typically arise from rapid movements that transfer some kinetic energy to the affected tissues, generating stresses that ultimately compromise the integrity of these structures. Accordingly, at face value, the IOC’s condition that a transfer of kinetic energy is a necessary condition for athletic injury occurrence may appear logically sound.

However, while this might seem like a compelling argument, it relies on inductive reasoning, generalising observed cases of common sports injuries to make a universal claim that kinetic energy transfer is necessary for all athletic injuries. This reasoning is not absolute; a single counterexample of an athletic injury occurring without a transfer of kinetic energy would successfully refute the claim, rendering it insufficient as a definitive foundation for understanding athletic injuries [8–11, 34, 39]. For example, a sustained load of high magnitude, such as those commonly encountered during weightlifting or arm wrestling, can result in tissue failures like bone fractures or tendon ruptures [107]. These scenarios arise from mechanical energy applied slowly or statically to a tissue, resulting in prolonged mechanical stress and creep deformation, and do not rely on either a rapid or a repetitive transfer of kinetic energy. Furthermore, according to the definitions provided by the WHO and ICD-11, a sunburn obtained during sports participation should also be classified as an injury, and this is caused by radiant energy.

To address this, one must probe at the fundamental essence of what an injury is [8, 40]. While deduction and induction are invaluable tools for disentangling and refining concepts, testing their coherence and logical independence to achieve greater precision and understanding, they are ultimately insufficient for apprehending the original essence of a concept [8, 40]. According to Aristotle, the essence of a concept must first be grasped through intellectual insight (*nous*) into its nature. Once this insight is achieved, it can then be named, defined, and assessed for coherence through logical demonstration (*Apodeixis*) [8, 39, 40, 108]. Indeed, just as the concept of ‘triangle’ cannot be used to prove that it means ‘triangle’, the concept of ‘injury’ cannot be used to prove that it means ‘injury’. Instead, its definition is rooted in its essence (*to ti ēn einai*, ‘what it is to be’), which must be taken as the starting point for any further analysis [8, 40]. The reasoning undertaken thus far in this article (e.g., disentangling pain and athlete availability from injury) was possible only by assuming both an implicit consensus about the general understanding of the concept of injury, and that the term injury signifies what an injury is, i.e., its definition [8]. These demonstrations are therefore inherently tautological in content (though indispensable in function) [19, 46]. So, what does an injury mean then?

A comprehensive list of current and historical definitions provided by various authoritative sources is presented in Table 4. The WHO and ICD-11, widely regarded as global authorities in health-related definitions and classifications, offer expanded definitions considered the gold standard for consistency and accuracy in health and medical sciences. As detailed earlier, the WHO defines injury as “a bodily lesion at the organic level” (Definition 2), and the ICD-11 describes it as “physical or physiological bodily harm” (Definition 3). A consistent theme across all provided definitions is that injury entails physical harm or damage to the body, encompassing physical hurt, lesions, and structural or physiological disruption. Such harm necessitates a disruption to the body’s physical structures and functions, which cannot occur spontaneously or in isolation; it *necessarily* requires a transfer of energy [109–111].

**Table 4:** (General) injury definitions

*Global authorities*	
World Health Organization (WHO) [30]	A bodily lesion at the organic level, resulting from acute exposure to energy (mechanical, thermal, electrical, chemical, or radiant), in amounts that exceed the threshold of physiological tolerance
Centers for Disease Control and Prevention (CDC) and The International Classification of External Causes of Injuries (ICECI) [84]	A (suspected) bodily lesion resulting from acute overexposure to energy (mechanical, thermal, electrical, chemical, or radiant) interacting with the body in amounts or at rates that exceed the threshold of physiological tolerance
International Classification of Diseases (ICD-11) [31]	Physical or physiological bodily harm resulting from interaction of the body with energy (mechanical, thermal, electrical, chemical or radiant, or due to extreme pressure) in an amount, or at a rate of transfer, that exceeds physical or physiological tolerance. Injury can also result from lack of vital elements, such as oxygen. Poisoning by and toxic effects of substances are included, as is damage of or due to implanted devices
*Top three leading medical dictionaries*	
Dorland’s Illustrated Medical Dictionary 33rd Edition (2019) [81]	Harm or hurt; usually applied to damage inflicted on the body by an external force. Called also trauma and wound
Stedman’s Medical Dictionary 28th Edition (2006) [82]	1. The damage or wound of trauma 2. Lesion
Taber’s Cyclopedic Medical Dictionary 25th Edition (2025) [83]	Injury [L. injuria, injustice] Blunt or penetrating trauma or damage to a part of the body SYMPTOMS: Various symptoms may occur, depending on the nature, extent, and severity of the damage. Mild injury produces pain, tissue swelling, redness, and temporary disruption of tissue function. Severe injury may result in irretrievable loss of the function of an organ, massive hemorrhage, or shock
*Generic dictionaries*	
Cambridge Dictionary (current) [112]	Physical harm or damage to someone’s body caused by an accident or an attack
Oxford Advanced Learners Dictionary (current) [113]	Harm done to a person’s or an animal’s body, for example in an accident
The Oxford Essential Dictionary of the U.S Military (2001) [114]	A condition of bodily damage. Injuries include fractures, wounds, sprains, strains, dislocations, concussions, and compressions. Conditions resulting from extremes of temperature or prolonged exposure, as well as acute poisonings (except those due to contaminated food) resulting from exposure to a toxic or poisonous substance are also classed as injuries
*Medical definitions through the ages*	
Dorland’s Illustrated Medical Dictionary 30th Edition (2003) [115]	Harm or hurt; usually applied to damage inflicted on the body by an external force. Called also trauma and wound
Dorland’s Illustrated Medical Dictionary 27th Edition (1988) [116]	Harm or hurt; a wound or maim; usually applied to damage inflicted on the body by an external force
Dorland’s Illustrated Medical Dictionary 24th Edition (1965) [117]	Harm or hurt; a wound or maim; usually applied to damage inflicted on the body by an external force
Dorland’s Illustrated Medical Dictionary (1941) 19th Edition [118]	Harm or hurt; a wound or maim
Dorland’s Illustrated Medical Dictionary 8th Edition (1915) [119]	Harm or hurt; a wound or maim
Stedman’s Medical Dictionary 23rd Edition (1976) [120]	Damage, wound, trauma
Stedman’s Medical Dictionary 6th Edition (1920) [121]	Damage, wound, trauma
Taber’s Cyclopedic Medical Dictionary 15th Edition (1985) [122]	Trauma or damage to some part of the body
Taber’s Cyclopedic Medical Dictionary 8th Edition (1961) [123]	A hurt or damage
Taber’s Cyclopedic Medical Dictionary 1st Edition (1940) [124]	A hurt or damage
An Illustrated Dictionary of Medicine, Biology and Allied Sciences 1st Edition (1894) [125]	Any damage or harm to the body or any of its parts
*Historical generic dictionaries*	
The Concise Oxford dictionary of Current English 1st Edition (1921) [126]	Harm, damage
Modern Dictionary of the English Language 2nd Edition (1911) [127]	Hurt or damage

Energy, whether mechanical, thermal, chemical, electrical, or other forms relevant to physical systems drives all physical change and is fundamental to the concept of injury. Without energy transfer, molecular bonds cannot be damaged, and no structural or functional changes can occur within the body. Ligaments cannot tear, bones cannot fracture, and cells cannot be damaged [109–111, 128]. It follows that physical or bodily harm, i.e., tissue damage, resulting from the transfer of energy, is the *to ti ēn einai* of injury, reflecting the essential properties necessarily tied to its identity [8, 34]. Indeed, how can an ACL or tendon injury exist, if the ACL or tendon has not been physically damaged? In the complete absence of a transfer of energy to a tissue and any resulting physical harm, an injury, by definition, cannot exist [30, 31]. To argue otherwise would be to claim that a broken leg, ACL rupture, or Achilles tendon tear receives the classification of an ‘injury’ not because the tissue has been physically damaged, but because of some other extrinsic property unrelated to the tissue itself, which is an absurd argument to make.

When considering this foundational understanding of injury, the IOC’s decision to limit the definition of athletic injury to those caused by kinetic energy is intriguing, as it diverges from the broader definitions proposed by the WHO and ICD-11. Injuries caused by the transfer of other energy forms besides kinetic, such as sunburn (radiant energy) or drowning (absence of energy), can certainly occur during sports participation. According to the WHO and ICD-11, these should be classified as injuries.

Ultimately, choosing a definition is a matter of ontological commitment, and reliance on authoritative definitions, when taken in isolation, can constitute an appeal to authority fallacy. Ontological or theoretical adequacy is not determined by institutional endorsement or consensus. Rather, on certain well-established philosophical accounts of scientific explanation, what justifies a concept as a legitimate scientific explanandum (Table 1) is coherent and sufficient mechanistic and causal unity: the requirement that the phenomena grouped under the concept are generated by the same underlying physical processes or causes and admit principled formalisation and falsifiable necessity claims [8, 10, 50, 59]. Accordingly, the authoritative definitions presented in Table 4 are not cited as proof of meaning in themselves, but as convergent evidence of a stable underlying essence that has been consistently recognised across historical and disciplinary contexts.

Mechanical injuries remain the most prevalent and consequential in sport, rendering the IOC’s definition of sports injury closely aligned with the primary objectives of sports medicine and injury research. This focus serves a practical purpose by distinguishing such injuries from forms of injury not typically understood as *athletic* or *sports* injuries, such as those caused by alternative energy sources (e.g., sunburn, chemical burns, electrical injuries) or the absence of energy (e.g., drowning, asphyxia). Nevertheless, should a broader scope be desired, expanding the definition of athletic injury presents no substantive linguistic or semantic difficulty. One straightforward solution would be to treat athletic injury not as a scientific explanandum, but as an umbrella term encompassing all injuries sustained in the context of sport, irrespective of energy source, thereby aligning with the broader frameworks adopted by the WHO and ICD-11. Within this structure, various subclasses exhibiting sufficient mechanistic unity to function as coherent scientific explananda [8, 10, 50, 59], such as mechanical, radiant, or thermal injuries, can be clearly delineated, preserving conceptual precision while enabling subclass-specific theorisation and modelling.

Relatedly, there have been increasing attempts to expand the general concept of injury to include additional subclasses, such as psychological injury [96, 129, 130]. If such inclusions are deemed necessary, this can be addressed by redefining injury at a higher level of abstraction and subsequently distinguishing physical and psychological injuries prior to further subclassification. This hierarchical approach maintains conceptual clarity and preserves mechanistic coherence while accommodating broader definitional commitments.

### Tissue Damage: Necessary but Insufficient

As the IOC restricts sports injury to the transfer of kinetic energy, bodily harm has been appropriately conceptualised as tissue damage. However, while tissue damage forms part of the *to ti ēn einai* of athletic injury, serving as a necessary condition for its existence, is the mere presence of tissue damage *sufficient* for an athletic injury to exist? Reasonably, no. This highlights a critical theoretical shortcoming in the definition proposed by the IOC (Definition 1); it fails to establish any reasonable sufficient conditions for an athletic injury to exist. To elaborate, tissue damage is an inevitable consequence of sporting participation [131–133], with even minor loading exposures resulting in some degree of tissue damage [134–136]. By adhering to the IOC’s definition, the quest for athletic injury prediction is over, as every athlete would inevitably incur an injury shortly after commencing their training, an outcome that is clearly unreasonable. Moreover, tissue damage often serves as a critical stimulus for tissue remodelling and adaptation [137–139], forming a normal part of the physical training and positive adaptation process. Consequently, equating the mere presence of tissue damage to an athletic injury sets an exceptionally low threshold for an athletic injury to occur, resulting in all athletes sustaining athletic injuries soon after engaging in sport.

An additional concern arises with the criterion of “other derangement of normal physical function” (Definition 1). Besides being overly vague, such derangements can occur without the presence of an injury. For instance, neuromuscular fatigue could be classified as a form of “other derangement of normal physical function “. Considering an athlete to have sustained an athletic injury as soon as they experience some degree of neuromuscular fatigue would similarly be unreasonable, while neuromuscular fatigue is also neither a necessary nor sufficient condition for an injury to exist, further demonstrating the logical shortcomings of this definition.

## Proposing a New Fundamental Theoretical Definition for Athletic Injury: Establishing the Sufficient Condition

*“There is no dividing line in nature, but only one drawn by the modeler, who chooses what to model”* – Bas van Fraassen paraphrasing John von Neumann [140]

Considering tissue damage is a necessary but insufficient condition for an athletic injury to exist, it follows (by definitional necessity for scientific coherence [2, 8–11, 13, 63]) that there must be some demarcating threshold of tissue damage that distinguishes an athletic injury from non-injury, which more closely reflects the definitions of (general) injury presented by the WHO (Definition 2) [30] and other notable organisations [31, 84] (Table 4). Accordingly, to address this, the following sufficient condition is proposed: the tissue damage sustained should not form part of the normal physical training and positive adaptation process but must exceed the threshold of mechanical and physiological tolerance. This is dependent upon the *nature* and *degree* of tissue damage sustained.

With the inclusion of this newly proposed condition for an athletic injury to exist, a new fundamental theoretical definition for athletic injury is presented:

“Tissue damage and loss of physical function during sports participation, resulting from singular, sustained, or repetitive transfer of mechanical energy, where the damage experienced is not a normal part of the physical training and positive adaptation process, but exceeds the threshold of mechanical and physiological tolerance.

This is dependent upon the nature and degree of tissue damage sustained” **(Definition 6).**

### Nature and Degree of Tissue Damage Sustained

Within the proposed definition (Definition 6), “*nature and degree of tissue damage sustained”* refers to the specific characteristics, properties, or type of tissue damage that distinguishes an athletic injury from normal responses to physical training. It encompasses both the qualitative aspect (e.g., the type of microstructures within specific tissue types affected) and the quantitative aspect (e.g., the extent or *severity* of the damage sustained).

An illustrative example highlighting the importance of considering the *nature* of tissue damage sustained is the distinction between muscle damage and muscle injury, which are distinct clinical entities [141]. In some contexts, muscle damage is a largely unavoidable and normal part of the physical training process [132, 133], often preceding beneficial adaptations such as the repeated bout effect [142], and increased hypertrophy and strength (although the causal nature of this relationship has been questioned [139, 143]). It is characterised by sarcomere dissolution and damage to various intramuscular microstructures, for example, desmin disruption and catabolism, Z-disk streaming, and other cytoskeletal or membrane alterations etc. [141, 144]. Given its frequent occurrence during and after training or competition [132, 133], and the beneficial adaptations that commonly ensue, reasonably, muscle damage should not be classified as an injury. Rather, muscle injury more accurately occurs when there are structural tears in muscle fibres [141], which provides no adaptive benefit and typically requires long and incomplete recovery processes [141].

The significance of considering the *degree* of tissue damage is exemplified by the distinction between the mechanical fatigue of bone and the development of bone cracks and fractures. Mechanical fatigue damage, characterised by a temporary reduction in bone stiffness and strength, is a stimulus for beneficial bone adaptation in accordance with Wolff’s Law [138, 145, 146]. In this context, the bone damage and microstructural changes that occur are part of a normal mechanical and physiological process that strengthens bone over time [138, 145, 146]. Conversely, the formation of macroscopic bone cracks or fractures due to excess damage represents a pathological outcome (i.e., the exceedance of mechanical and physiological tolerance, and therefore an injury in this context), resulting in prolonged losses in bone density and strength, ultimately compromising bone health [147].

### Singular, Sustained, or Repetitive Transfer of Mechanical Energy

Within Definition 6, “*rapid or repetitive transfer of kinetic energy*” has been replaced with “singular, sustained, or repetitive transfer of mechanical energy”. This change is proposed as a more accurate and inclusive approach, with mechanical energy encompassing kinetic energy while also accounting for injuries resulting from sustained high-magnitude loads, such as those encountered in weightlifting. It is agreed, however, to exclude other forms of energy (e.g., radiant, electrical, chemical). While such injuries may occur during sports participation, these are not typically what is meant by an ‘athletic’ injury. Currently, extensive evidence demonstrates that mechanically induced tissue damage reflects the damage profiles observed in sports-related injuries [107, 134, 135, 144, 148–153]. Even in complex active tissues like muscle, mechanical loads are essential for causing fibre or musculo-tendinous ruptures [153, 154]. Another distinct alteration from the definition proposed by the IOC (Definition 1) is the omission of the phrase “other derangement of normal physical function“. This phrase has been excluded for two reasons: it is overly vague, and, as highlighted in the neuromuscular fatigue example presented in Sect. 3.3, it is neither necessary nor sufficient for an athletic injury to exist.

### Loss of Physical Function

In the proposed definition (Definition 6), “*loss of physical function”* refers to the objectively measurable deterioration in a tissue’s mechanical properties, such as load-bearing capacity, stiffness, and elasticity. While this phenomenon might not strictly belong in the fundamental definition of injury, since it does not constitute the essence itself, it nonetheless represents a property that necessarily follows from it. In Aristotelian terms, it may therefore be considered an *idion* (Table 1) rather than a *symbebekós*: a proper attribute that, though not part of the essence, accompanies it necessarily. Its inclusion is justified not only by its theoretical coherence within the underlying framework but also by its value for operationalisation, as it bridges the theoretical definition with measurable indicators. As these mechanical properties are fundamentally governed by molecular bonding, tissue damage (i.e., damage to molecular bonds maintaining tissue integrity) necessarily compromises them at localised levels. While these relationships may appear disassociated at higher structural levels, this is a function of scale and emergent behaviour, such as stress redistribution and deformation (discussed further in Sect. 4.4). Accordingly, loss of physical function is not viewed in isolation but as an *idion* of tissue damage within a unified physics-based framework. This allows their relationship to be mathematically defined and modelled, providing a precise understanding of how tissue damage impairs functional capacity.

### Operationalisation: Physical Manifestation of Tissue Damage and Mathematisation of Athletic Injury

*“The earlier view, that for some terms of theoretical vocabulary there could be definitions in terms of the observational vocabulary, called either ‘correlative definitions’ (Reichenbach) or ‘operational definitions’ (Bridgman), has been abandoned by most empiricists as an oversimplification… The terms of the theoretical vocabulary obtain only an indirect and incomplete interpretation by the fact that some of them are connected by the corresponding rules with observational terms”* – Rudolf Carnap [12]

*“The sciences do not try to explain, they hardly even try to interpret, they mainly make models. By a model is meant a mathematical construct which, with the addition of certain verbal interpretations, describes observed phenomena. The justification of such a mathematical construct is solely and precisely that it is expected to work— that is, correctly to describe phenomena from a reasonably wide area”* – John von Neumann [25]

While the practical implications of the newly proposed theoretical definition of athletic injury (Definition 6) will be explored in detail in other works, several important considerations are highlighted here. Central to the proposed definition of athletic injury is its emphasis on tissue damage (Table 5), which, while constituting part of the necessary essence of the concept, has the added benefit of enabling operational definitions that can be aligned with objective criteria. This alignment minimises the influence of human perception and decision making, reducing bias and supporting more consistent understandings of athletic injury. For example, if bone injury (a subcategory of *athletic injury*) is operationalised as the onset of cracking (or a certain degree of cracking), this is not reliant upon subjective bias but can be objectively assessed and modelled. Such objectivity enhances the testability and reproducibility of athletic injury research, while also facilitating the development of more sensitive measurement tools [10].

**Table 5:** Mechanical and modelling foundations of tissue damage and failure

*Material and tissue behaviour*	
Tissue damage	Tissue damage refers to the disruption of the molecular bonding that maintains tissue integrity and functional capacity, resulting from the transfer of energy, such as mechanical, radiant, thermal, chemical, or electrical energy. However, while this bond-level damage plays a central causal role in tissue degeneration and failure, in the absence of overt manifestations of structural disruption (e.g., cracking or tearing), tissue damage is commonly unobservable. Instead, tissue damage must be inferred from indirect indicators such as measurable changes in tissue-level mechanical properties or imaging markers Notably, the physically observable manifestations of tissue damage vary by tissue type. For example, microcracks, diffuse damage, and cracking in bone [148]; collagen unfolding, kinked fibres, and tearing in tendons [149, 150]; and sarcomere disruption and fibre tears in muscles [141, 144]
*Mechanical fundamentals*	
Mechanical loading	Mechanical loading refers to the external force or combination of forces applied to a tissue, causing stresses and strains. Depending on the nature and direction of the applied forces, loading can come in a variety of modes e.g., tension, compression, shear, bending, or torsion
Mechanical stress	Stress is defined as the internal force per unit area that develops within a tissue in response to an applied force. Stress may be characterised as normal (force perpendicular to a plane) or shear (force parallel to a plane) Normal stress may be tensile or compressive depending on the mode of loading
Mechanical strain	Strain is a normalised measure of tissue deformation expressed as the ratio of deformation to the initial dimensions. Two types of strain exist: normal strain, which is related to changes in size, and shear strain, which is related to changes in shape. Normal strain may be tensile or compressive depending on the type of loading
*Modelling frameworks for physical damage and failure*	
Finite element modelling	Finite element modelling is a numerical method used to approximate solutions to complex physical problems by dividing a structure or system into smaller, simpler parts called finite elements. These elements are connected at discrete points (nodes), and mathematical equations are applied to simulate how the system responds to forces, stresses, or other physical phenomena
Continuum damage mechanics	Continuum damage mechanics is a theoretical framework used to model and predict the initiation and progression of material damage. It describes the gradual degradation of material properties, such as stiffness and strength, through the use of damage variables that represent the accumulation of microscopic defects, like cracks or voids, within the material

Additionally, tissue damage and athletic injury can be formalised for application in mathematical modelling (e.g., prediction, simulation, and causal inference). To elaborate, in mechanical models quantifying the accumulation of damage over time, typically arising from mechanical loading and the resulting stresses and strains (Table 5), damage is commonly represented using a damage variable (*D*) ranging between 0 and 1, where (*D* = 0) corresponds to an undamaged state and (*D* = 1) corresponds to complete mechanical failure, i.e., an inability to carry load [136, 155, 156].

Adopting a similar approach, athletic injury can be formalised and mathematically defined as:

*D* > *D*_*c*_** (Definition 7)**

In Definition 7, first proposed by Edwards [136], an athletic injury occurs when the damage (*D* – quantified between 0 and 1) sustained by a tissue is greater than a critical damage threshold (*D*_*c*_ – also quantified between 0 and 1), i.e., *D* > *D*_*c*_. To provide an example of this, the formation of cracks (or a certain degree of cracking) in bone would be represented by a specific damage threshold, allowing for its prediction within mathematical models. Ultimately, however, the observable physical manifestation of damage varies between tissues, for example, microcracks, diffuse and cracking in bone [148], kinked fibres and tearing in tendon [149], sarcomere disruption and fibre tears in muscle [141, 144], etc. and accordingly, consideration of the tissue-specific manifestations of damage are ultimately needed.

Importantly, in the absence of overt and measurable physical damage such as cracking or tearing, tissue damage must be inferred from measurable changes in mechanical properties. This links microscopic damage to observable functional impairments. In most engineering settings, damage is commonly quantified and modelled by assessing the degradation of load-bearing capacity, stiffness characteristics, and deformation patterns (e.g., strain or creep) [136, 157]. Such approaches consistently demonstrate high predictive validity for observable phenomena, including crack formation, propagation, and structural failures – including in human tissues [149, 157, 158]. However, while load-bearing capacity certainly warrants special consideration, determining whether a tissue ultimately fails, a tissue’s role may extend beyond load bearing to include functional tasks such as storing and releasing energy to drive locomotion. Accordingly, other mechanical properties, such as elasticity and hysteresis, should also be considered [136, 157].

At the structural level, the relationship between localised damage and mechanical properties may become decoupled. However, this is a function of scale, due to scale-dependent emergent behaviours such as stress redistribution and deformation. To address this complexity and improve predictive precision, practical engineering approaches such as finite element modelling and continuum damage mechanics (Table 5) account for these effects by modelling how localised damage influences macroscopic mechanical behaviour [159, 160].

Finally, while traditional mechanical models modelling fatigue damage accumulation commonly determine damage accumulation based on the mechanical loading pattern experienced by a structure or material [105, 136, 161], in the context of athletic injury, which involves biological tissues, damage includes both damage due to loading and any alterations in damage induced by physiological processes, such as remodelling and repair [105, 136, 162]. This is particularly relevant to athletic injuries exhibiting a gradual onset mechanism, whereby significant damage removal can occur during periods of rest and recovery [163, 164].

### The Setting of a Critical Tissue Damage Threshold

Identifying relevant critical damage thresholds that are reflective of the proposed theoretical definition for athletic injury is a difficult task. In materials sciences, such thresholds are typically determined through material testing protocols, which commonly involve the application of various forms of stress (either singular or repetitive) to assess the mechanical behaviour of a material under load. Through this process, relevant thresholds can be determined based on empirical evidence supporting the applicability of a particular threshold relative to the intended use or function of a structure or material. Then, in practice, this threshold can be estimated using predictive models.

In applied sporting scenarios, selecting an exact critical damage threshold may, in some contexts, be a challenging endeavour, with such a threshold likely relying upon the triangulation of empirical evidence to support its relevance. For instance, this may involve an analysis surrounding the degradation of physical function, i.e., mechanical properties such as stiffness, elasticity, and strength, that commonly accompany damage accumulation, the observable physical manifestation of damage (which varies between tissues, e.g., cracking in bone [148], kinked fibres in tendon [149] etc.), and the future recovery and remodelling of the tissue, i.e., the adaptations that ensue. In certain scenarios, the setting of a relevant threshold may be relatively straightforward. For example, the physical manifestation of tissue damage, such as the development of cracks (or a certain degree of cracking) in bone, may serve as an appropriate tissue injury threshold, with this physical manifestation corresponding with a particular mathematical tissue damage threshold between 0 and 1. If a researcher is interested in complete tissue failure, such as tendon rupture or bone fracture, the damage threshold can be set to 1, which also represents the exceeding of the failure strength of a tissue and a complete loss of functional capacity, i.e., a complete inability to tolerate load. Ultimately, however, due to the considerable differences that exist between tissue types, precise operationalisations tailored to specific tissue types (i.e., defining critical damage thresholds for bone, tendon, muscle, etc.) are needed.

### Scale-Dependence and Invariance of the Injury Threshold Condition (D > D_c_)

It is important to note that the condition *D* > *D*_*c*_ is scale dependent. Accordingly, the empirical interpretation of ‘injury’ varies within the framework according to the spatial scale of analysis, that is, the structural level to which the condition is applied. At localised or microscopic levels, this condition may represent discrete regions of material failure (e.g., broken collagen fibrils or trabecular microcracks) that might not substantially impair whole-structure function. When applied at the whole-structure level, *D* > *D*_*c*_ should not be interpreted as macroscopic failure (which corresponds to *D* = 1), but rather a mathematical construct inferred from measurable changes in mechanical properties that mark the transition from a non-injured to an injured state within the theoretical framework. In this sense, it serves as a predictive demarcation, linking the degree of tissue damage to functional degradation, risk of failure, or recovery trajectories within the model. This predictive capacity across scales highlights that, while the manifestation of injury may vary, the definition of injury as damage exceeding a critical threshold of integrity (i.e., *D* > *D*_*c*_) remains invariant, expressing the same law-like constitutive principle at every level of structural organisation and preserving theoretical coherence across hierarchical levels of biological organisation. Furthermore, any selected threshold can be used to model relationships between injury and its derivatives (explored in the following section), as well as with other constructs of interest such as time-loss or performance decrements.

## Developing a Formalised and Mathematised Ontological Framework for Modelling Injury and Associated Concepts: Foundations and Initial Expansions

*“Every kind of science, if it has only reached a certain degree of maturity, automatically becomes a part of mathematics”* – Common paraphrase of David Hilbert [165]

*“If we have correspondence rules for certain terms, and these terms are connected with other terms by the postulates of theory, then these other terms thereby also acquire observational significance.”* – Rudolf Carnap [12]

By mathematically defining injury as a state in which tissue damage (*D*) exceeds a critical damage threshold (*D* > *D*_*c*)_, the foundation is established for the development of a formalised and mathematised ontological framework suitable for application in mathematical modelling (e.g., prediction, simulation, and causal inference). This framework provides a logically coherent structure that systematically integrates concepts associated with injury, such as severity and recovery, transforming them from vague and inconsistently used notions into measurable and predictable quantities governed by precise mathematical relationships. In the Carnapian sense [12], these constructs acquire partial empirical meaning through their interrelations within the formal system and their correspondence to observable and measurable physical parameters, such as tissue stiffness, strength, deformation, and the progression or reduction of lesions [12]. These correspondence rules connect the emerging theoretical framework of injury to observable phenomena, ensuring that its mathematical formulation remains empirically interpretable [12]. For example, injury severity (Sev), i.e., the extent of injury, naturally follows as the degree to which damage surpasses the critical damage threshold, mathematically expressed as:

1 $${\text{If }}D{ > }D_{c} {\text{, then Sev = }}D - D_{c} .$$

Severity can then be rescaled and normalised to be expressed as a quantity between 0 and 1, where 0 represents the complete absence of injury severity, corresponding to a non-injured state (*D* ≤ *D*_*c*_), and 1 represents complete tissue failure, corresponding to maximal injury severity (Sev = 1 ⟺ *D* = 1). This rescaling can be expressed as:

2 $$D>{D}_{c},\text{ then Sev}=\frac{{\boldsymbol{D}}-{\boldsymbol{D}}{\boldsymbol{c}}}{1-{\boldsymbol{D}}{\boldsymbol{c}}}.$$

Importantly, severity does not exist when injury does not exist. That is, when $D\le {D}_{c}$, the tissue remains uninjured and severity is undefined.

Tissue recovery (*R*) can be defined as a reduction in tissue damage, expressed mathematically as:

3 $${\text{If }}D_{t1} { > }D_{t2} {\text{, then }}R{ = }D_{t1} - D_{t2} ,$$

Where *D*_*t*1_ represents the level of tissue damage at an initial time point, *D*_*t*2_ represents the level of tissue damage at a later time point, and *R* quantifies the amount of damage removed between *D*_*t*1_ and *D*_*t*2_, assuming *D*_*t*2_ < *D*_*t*1_. If *D*_*t*2_ ≥ *D*_*t*1_, no recovery has occurred, and if *D*_*t*2_ > *D*_*t*1_, additional damage has been accumulated.

Recovery can then also be rescaled and normalised to range between 0 and 1, where 0 represents no recovery (i.e., no tissue damage removal) and 1 represents complete recovery corresponding to a return to an undamaged state (*R* = 1 ⟺ *D* = 0). This normalised form can be expressed as:

4 $${\text{If }}D_{t1} \ge D_{t2} {\text{, then }}R{ = 1} - \, \frac{{D_{t2} }}{{D_{t1} }}.$$

This scaling allows *R* to function as a percentage, where *R* = 0 corresponds to 0% recovery (i.e., no damage removal) and *R* = 1 corresponds to 100% recovery (i.e., complete restoration to an undamaged state). Intermediate values between 0 and 1 reflect the proportion of recovery achieved.

Rate of recovery (*Ṙ*) can then be defined as the amount of damage removed over a defined time period and can be mathematically expressed as:

5 $${\text{If }}D_{t1} { = }D_{t2} {\text{, then (}}\dot{R}{) = }\frac{{D_{t1} - D_{t2} }}{{t_{2} - t_{1} }},$$

where *D*_***t*1**_ represents the level of tissue damage at an initial time point (*t*1), *D*_***t*2**_ represents the level of tissue damage at a later time point (*t*2), and *Ṙ* quantifies the amount of damage removed between *D*_*t1*_ and *D*_*t2*_ over the defined time period (*t2*–*t1*), assuming *D*_***t2***_ ≤ *D*_***t1***_. If *D*_***t2***_ ≥ *D*_***t1***_, no recovery has occurred. If *D*_***t2***_ > *D*_***t1***_**,** additional damage has been accumulated.

Recovery from injury (*R*_injury_) can be defined as the reduction in tissue damage relative to the injury threshold and can be expressed as a function of injury severity as follows:

6 $${\text{If }}D{ > }D_{c} {\text{, and Sev}}_{t1} {\text{ = Sev}}_{t2} {\text{, then }}R_{{{\mathrm{injury}}}} {\text{ = Sev}}_{t1} - {\text{ Sev}}_{t2} ,$$

*w*here Sev_*t1*_ represents the level of injury severity at an initial time point, Sev

_*t2*_ represents the level of injury severity at a later time point, and *R*_injury_ quantifies the reduction in injury severity between these time points, assuming Sev_*t1*_ > Sev_*t2*_. If Sev_*t1*_ ≤ Sev_*t2*_, no recovery from injury has occurred. If Sev_*t2*_ > Sev_*t1*_, additional damage has been accumulated and injury severity has increased.

Recovery from injury can also be rescaled and normalised to range between 0 and 1, where 0 represents no recovery from injury (i.e., no removal of tissue damage), and 1 represents a return to an uninjured state. This can be expressed as:

7 $${\text{If }}D_{t1} \ge D_{t2} \ge D_{c} {\text{, then }}R_{{{\mathrm{injury}}}} { = }\frac{{D_{t1} - D_{t2} }}{{D_{t1} - D_{c} }},$$

where *D*_*t*1_ represents the level of damage at an initial time point, *D*_*t*2_ represents the level of damage at a later time point, and *D*_*c*_ represents the critical damage threshold distinguishing injury from non-injury. This scaling allows recovery from injury to function as a percentage, where *R*_injury_ = 0 corresponds to 0% recovery from injury (i.e., no damage removal) and *R*_injury_ = 1 corresponds to 100% recovery from injury, defined as a return to a non-injured state (*D* ≤ *D*_*c*_).

The rate of recovery from injury (*Ṙ*_injury_) can then be defined as the rate at which injury severity decreases and can be expressed mathematically as:

8 $$\dot{R}_{{{\mathrm{injury}}}} = \frac{{{\mathrm{Sev}}_{1} - {\mathrm{Sev}}_{2} }}{{t_{2} - t_{1} }},$$

where Sev_*t1*_ represents the level of injury severity at an initial time point (*t*1), Sev_*t*2_ represents the level of injury severity at a later time point (*t*2), and *Ṙ*_injury_ quantifies the reduction in injury severity over the time interval (*t*2−*t*1), assuming Sev_*t*2_ < Sev_*t*1_. If Sev_*t*2_ ≥ Sev_*t*1_, no recovery from injury has occurred, and if Sev_*t*2_ > Sev_*t*1_, additional damage has been accumulated.

While the further expansion and utilisation of this ontological framework and semantic network will be addressed in more comprehensive future works, its value should already be evident in the marked increase in conceptual precision. Each concept is introduced within a logically consistent system and defined through precise mathematical relationships, all coherently linked to the foundational definition of injury (*D* > *D*_*c*_) and its derivatives. These expansions establish a structured network where concepts such as injury, severity, recovery, rate of recovery, and adaptation collectively contribute to a comprehensive and mathematically precise understanding of the overarching system of injury.

### Limitations of the Mathematical System

While the mathematical framework developed in this article is grounded in well-established principles of continuum damage mechanics [156, 157, 160, 166], which supports its validity, several important limitations should be recognised. As molecular-level damage is not currently observable, the framework necessarily uses a damage variable (*D*) to represent the accumulation of damage and loss of tissue integrity. Accordingly, when applied at the structural level it necessarily simplifies the complex and heterogeneous nature of biological tissues, which may exhibit different damage behaviours (e.g., viscoelastic, plastic, or brittle failure) across structural layers. Importantly, however, this limitation can be mitigated by applying the same mathematical logic to smaller, localised regions within a tissue. This approach retains the underlying theoretical coherence of the system while enabling higher-resolution modelling and greater empirical precision as data become available. In this way, the framework remains both conceptually robust and adaptable to progressive refinement, which is a hallmark of effective scientific modelling.

## Summary of Key Contributions and Conceptual Advancements in this Article

To consolidate the key contributions of this article, Table 6 summarises the major theoretical, logical, and mathematical advancements presented. It integrates the central developments across the paper’s stages of analysis and highlights how the new theoretical definition of athletic injury helps align the theoretical, observational, and mathematical dimensions of the construct, thereby establishing the foundation for its formalisation within a coherent theoretical system and its application in predictive modelling.

**Table 6:** Summary of key contributions and conceptual advancements in this article

Clear, well-defined concepts are essential for scientific inquiry, enabling effective theory formation and operational precision, while also upholding the core scientific principles of predictability, testability, falsifiability, and reproducibility. Athletic injury remains inadequately conceptualised and is often used in vague or contradictory manners, obscuring theoretical clarity, undermining modelling efforts, and eroding predictive accuracy
To address this, a new theoretical definition of athletic injury was proposed, developed through a systematic process of Carnapian explication and metaphysical inquiry, grounded in the principles of Aristotelian essentialism and classical logic. This approach leveraged well-established logico-philosophical tools such as thought experiments, boundary tests, and logical reasoning, to disentangle conflated concepts and establish necessary and sufficient conditions for an athletic injury to exist
Through this process, commonly conflated concepts (*Symbebekós*, ‘accidental properties’) were examined for logical independence and disentangled, and the development of a more refined conceptualisation and definition of athletic injury was achieved, capturing its fundamental essence (*To ti ēn einai*, ‘what it is to be’) as: *“Tissue damage and loss of physical function during sports participation, resulting from singular, sustained, or repetitive transfer of mechanical energy, where the damage experienced is not a normal part of the physical training and positive adaptation process, but exceeds the threshold of mechanical and physiological tolerance. This is dependent upon the nature and degree of tissue damage sustained.”*
By introducing a demarcating threshold of tissue damage and loss of physical function to distinguish athletic injury from non-injury, this definition provides the sufficient condition for an athletic injury to exist, and aligns this construct more closely with the definitions of (general) injury proposed by the World Health Organization (WHO) and International Classification of Diseases (ICD-11). Furthermore, this clarification provides the necessary conceptual and logical foundations for scientific formalisation and operationalisation
While an injury may be best represented as a lesion at the clinical or diagnostic level – as reflected in definitions of injury from the World Health Organization (WHO) and Centers for Disease Control and Prevention (CDC) – for the purposes of predictive modelling, simulation, and causal inference, athletic injury is more appropriately operationalised as a logico-mathematical construct. Here, athletic injury is defined as occurring when the damage (*D*) experienced by a tissue exceeds a critical damage threshold (*D*_*c*_), i.e., *D* > *D*_*c*_
In the case of *time-loss injuries*, the time-loss injury construct sacrifices empirical testability and reproducibility in favour of operational convenience. Accordingly, for the construct to function in a scientific rather than merely administrative capacity, namely, as something that can be reliably predicted, *D* > *D*_*c*_ must remain a prerequisite for its existence. Without tissue damage exceeding a critical threshold, there is no reliable basis on which to distinguish genuine injury cases from other forms of absence, such as low motivation, strategic withdrawal, or faking an injury. In other words, without anchoring this construct to the modelling of the underlying tissue-damage state, the time-loss injury construct collapses into a generic measure of athlete availability rather than injury, eroding both conceptual integrity and predictive value
While the formation of lesions represents the observable manifestation of tissue damage surpassing a critical damage threshold at localised scales, the damage variable (*D*) is a latent variable that cannot be directly observed. Instead, *D* must be inferred from objectively measurable physical parameters *(‘loss of physical function’)*, such as tissue mechanical strength, stiffness characteristics, and deformation patterns (e.g., strain or creep). These serve as proxy indicators of the underlying damage state and can be integrated into probabilistic models for assessing and predicting tissue damage accumulation, lesion formation and progression, tissue failures, and injury risk
This definition also establishes the foundation for a formalised and mathematised ontological framework and semantic network surrounding athletic injury. Within this framework, athletic injury, and related constructs such as injury severity, recovery, and rate of recovery, are systematically defined through precise mathematical relationships, each coherently linked to the fundamental condition *D* > *D*_*c*_. In the Carnapian sense, these constructs acquire partial empirical meaning through their interrelations within the formal system and their correspondence to measurable physical parameters (e.g., tissue stiffness, deformation, or strength). Their empirical meaning thus arises not from direct observation, but from their position within a logically coherent, mathematised structure that connects theory and observation
Finally, this article represents a significant advancement in the theoretical and scientific understanding of athletic injury. Through the process of explication, an imprecise, pre-scientific notion has been transformed into a coherent and precisely defined construct suitable for scientific use. In doing so, athletic injury has been clarified and integrated into a formal logico-mathematical framework, enabling it to better satisfy the core criteria required of a scientific concept. The result is not merely a refined definition but an *explicated construct* that connects theoretical reasoning, empirical observation, and mathematical formalism within a single, logically consistent system. This work therefore establishes a unified and scientifically rigorous foundation for the study, modelling, and prediction of athletic injury and associated constructs

## Conclusion

This article has introduced a new theoretical definition of athletic injury, developed through a systematic process of Carnapian explication and logical reasoning. By identifying necessary and sufficient conditions, commonly conflated concepts (*symbebekós*) have been successfully disentangled, and the conceptual and logical shortcomings of existing definitions have been resolved, capturing the fundamental essence (*to ti ēn einai*) of athletic injury as:

“Tissue damage and loss of physical function during sports participation, resulting from singular, sustained, or repetitive transfer of mechanical energy, where the damage experienced is not a normal part of the physical training and positive adaptation process, but exceeds the threshold of mechanical and physiological tolerance.

This is dependent upon the nature and degree of tissue damage sustained.”

The development of a conceptually and logically coherent theoretical definition provides an important foundation for enhancing the predictability, testability, falsifiability, and reproducibility of athletic injury as a scientific concept, enabling its formalisation and operationalisation as a logico-mathematical construct (i.e., *D* > *D*_*c*_), suitable for prediction, simulation, and causal inference. This transformation has established the basis for a formalised and mathematised ontological framework and semantic network surrounding athletic injury, offering a structured mathematical architecture that integrates related constructs such as severity, recovery, and rate of recovery within a coherent theoretical system. This objective framework transforms athletic injury and its associated constructs from vague notions, subject to inconsistent interpretation and application, into logically consistent mathematical objects with well-defined semantics and well-founded logic.

Finally, by explicitly aligning the theoretical (definitional and logical), observational (lesion formation and progression, mechanical degradation, tissue failures), and mathematical (formal modelling) dimensions of athletic injury, this framework bridges conceptual understanding with empirical measurability and analytical precision. This integration ensures that the theoretical definition is not merely philosophical but empirically anchored and mathematically actionable. Ultimately, this marked increase in conceptual precision and dimensional alignment will facilitate advancements in assessment technologies, data analysis, and model development, improving the identification, measurement, and prediction of athletic injury and related phenomena.
